# A new twist on lactate signaling: alanyl-tRNA synthetases 1 and 2 as metabolic sensors and lactyltransferases

**DOI:** 10.1186/s43556-025-00296-1

**Published:** 2025-08-23

**Authors:** Ting Xiao, Fubing Wang, Xinghua Long

**Affiliations:** Department of Laboratory Medicine, Zhongnan Hospital of Wuhan University, Wuhan, China

A recent study published in *Nature* [1] by Li et al. revealed that alanyl-tRNA synthetases 1 and 2 (AARS1/2) are L-lactate sensors, with AARS2 responding to L-lactate and regulating cyclic GMP-AMP synthase (cGAS) activity via lysine lactylation (Kla). This discovery provides new insights into the modulation of immune responses by metabolic cues and offers potential therapeutic avenues for immune and inflammatory diseases.

One of the important metabolic characteristics of tumor cells is their tendency to use aerobic glycolysis to metabolize glucose, which produces large amounts of lactate, a process known as the Warburg effect. While traditional views have considered lactate to be a metabolic waste product, recent studies have shown that lactate plays a complex and important role in tumor progression and immune regulation as a key metabolite in the tumor microenvironment. For example, lactate accumulation affects tumor cell signaling pathways, and the interaction between lactate and immune cells also affects cell differentiation, immune response, immune surveillance and sensitivity to therapy [2]. Additionally, Zhang et al. identified Kla as a novel post-translational modification and demonstrated that lactate-derived histone Kla modifications regulate macrophage polarization, revealing a direct molecular link to the epigenetic regulation of immunity [3]. Nevertheless, the enzymatic basis of Kla remains a fundamental unresolved question in the field.

Li et al. demonstrated that human cytomegalovirus (HCMV)-infected patients with normolactatemia exhibited significantly elevated 2′3'-cGMP-AMP (cGAMP) and IFNβ levels compared to hyperlactatemic or lactic acidosis cohorts, with strong inverse correlations observed between these immune mediators and serum L-lactate concentrations [1]. Intracellular L-lactate pools derive from both lactate dehydrogenase A (LDHA)-dependent synthesis and monocarboxylate transporter 1 (MCT1)-mediated uptake. Strikingly, murine peripheral blood mononuclear cells and bone marrow-derived macrophages displayed minimal intrinsic L-lactate production, while MCT1 inhibition via ARC155858 (ARC) or AZD3965 (AZD) blocked intracellular L-lactate accumulation. Both inhibitors fully rescued herpes simplex virus type 1 (HSV-1)-suppressed cGAMP synthesis and IFNβ expression after NaLac pretreatment, implicating MCT1 as a critical regulator of lactate-driven immune evasion. Next, peritoneal macrophages from bone marrow cell-specific *Mct1* knockout mice (*Lyz2-cre*^+^*Mct1*^*fl/fl*^) exhibited significantly enhanced HSV-1-induced cGAMP production and IFNβ expression compared to *Lyz2-cre*^*−*^*Mct1*^*fl/fl*^ [1]. However, NaLac or L-lactate pretreatment in *Lyz2-cre*^*−*^*Mct1*^*fl/fl*^ macrophages suppressed cGAMP and IFNβ expression while increasing HSV-1 titers. Similarly, NaLac pretreatment in wild-type mice reduced cGAMP and IFNβ expression while increasing HSV-1 viral loads, correlating with enhanced viral susceptibility. But these effects were not found in *Lyz2-cre*^+^*Mct1*^*fl/fl*^. Li et al. not only revealed that MCT1-mediated L-lactate uptake inhibits cGAMP synthesis and innate immune surveillance, but further experiments also demonstrated that intracellular L-lactate can be sensed and catalyze the lactylation of cGAS, which then inactivates cGAS.

Through genome-wide gRNA library screening, Li et al. ascertained *AARS1/2* as key regulators of sense intracellular L-lactate and cGAS inactivation. A global lysine lactylome analysis demonstrated that *AARS1/2* knockdown attenuated L-lactate-induced signaling, while ectopic expression increased proteome-wide Kla. Mechanistically, AARS1/2 catalyze lactylation via a two-step ATP-dependent process: L-lactate activation to lactate-AMP with PPi release, followed by L-lactate transfer to target protein lysine residues with AMP liberation. This work establishes AARS1/2 as bifunctional lactate sensors and lactyltransferases. Interestingly, beyond its canonical role in protein translation, AARS1 exhibits moonlighting activity as a lactate transferase, mediating lactylation modifications that couple metabolic reprogramming with proteomic alterations to drive tumorigenesis [4].

The cGAS-stimulator of interferon genes (STING) signaling pathway serves as an important monitor of the widespread response to tissue injury and pathogen invasion, and its aberrations trigger a variety of human diseases, including infectious diseases, autoimmune diseases, and tumors. It detects cytoplasmic DNA or DNA damage via the DNA sensor cGAS, and the resulting cGAMP activates STING, which can activate transcription factors such as IFN regulatory factor 3 (IRF3) to enter the nucleus and orchestrate a broad spectrum of antiviral responses, including expression of type I IFN [5]. This process couples DNA sensing with innate immune defense. Li et al. reveal the modulatory effect of lactylation on cGAS. L-Lactate sensor AARS2 upon sensing L-lactate mediates the N-terminal lactylation of cGAS, inverting its function and inhibiting cGAMP synthesis. Inhibition of cGAMP synthesis by cGAS lactylation does not significantly activate STING, which activates the transcription factor IRF3 to enter the nucleus and coordinate the antiviral response.

Innovationally, Li et al. developed a genetic code expansion system to site-specifically introduce lactate modifications on cGAS, enabling precise functional characterization (Fig. 1). Their analyses revealed that lactylation reduces cGAS-DNA binding affinity, as unlactated modified cGAS (cGAS(non-Lac)) formed larger, more reversible liquid droplets with short DNA compared to lactylated cGAS (cGAS(Lac)). Moreover, especially together with long DNA, cGAS(non-Lac) demonstrated enhanced cGAMP synthesis and efficient liquid-like phase separation (LLPS), while cGAS(Lac) exhibited impaired enzymatic activity and DNA exclusion, forming cGAS(Lac)-DNA void-rich dimeric droplets. These findings were corroborated in vivo, where N-terminal Kla abrogated cGAS-mediated DNA sensing. Elevated serum L-lactate levels promoted mitochondrial DNA release through impaired granulocyte ATP metabolism, with lactylated cGAS exhibiting defective self-DNA recognition. Furthermore, Li et al. identified an anxiety-induced immune suppression pathway mediated by cGAS lactylation, which was reversible upon MCT1 inhibition. These findings elucidate a molecular mechanism underlying immune dysregulation in anxiety disorders and suggest MCT1 blockade as a potential therapeutic intervention.

**Fig. 1:**
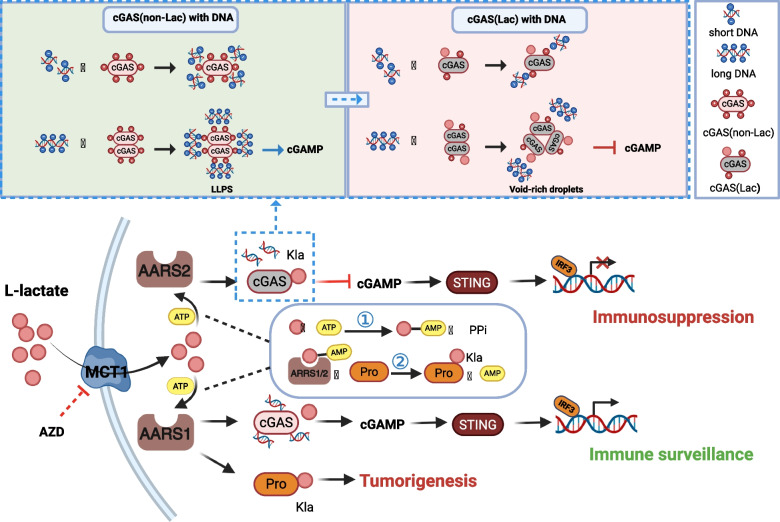
AARS1/2 function as a lactate sensor and lactyltransferases. L-lactate is introduced intracellularly via MCT1, which can be sensed by AARS1/2 and, in a two-step process, transfer L-lactate to lysine residues of target proteins. This process can be blocked by AZD. This study revealed that lactylation reduces cGAS-DNA binding affinity, as unmodified cGAS(non-Lac) formed larger, more reversible liquid droplets with short DNA compared to cGAS(Lac). Especially together with long DNA, cGAS(non-Lac) demonstrated enhanced cGAMP synthesis and efficient LLPS with long DNA, while cGAS(Lac) exhibited impaired enzymatic activity and DNA exclusion. AARS1 cannot mediate the lactylation of cGAS, and cGAS can still exercise an immunosurveillance function. However, it has been demonstrated that AARS1 lactylates other proteins and thus promotes tumorigenesis. Meanwhile, AARS1/2 are ATP-dependent lactyltransferases and catalyze protein lactylation in a two-step reaction. MCT1: monocarboxylate transporter 1; AZD: AZD3965 (a MCT1 inhibitor); ATP: Adenosine triphosphate; AMP: Adenosine monophosphate; PPi: pyrophosphate; Kla: lysine lactylation; Pro: protein; cGAS: cyclic GMP-AMP synthase; STING: stimulator of interferon genes; IRF3: IFN regulatory factor 3; cGAS(non-Lac): unlactated modified cGAS; cGAS(Lac): lactylated cGAS. (created in https://BioRender.com)

Li et al.'s study is notable for its identification of enzymes with dual functions, broadening our understanding of how metabolic changes affect immune signaling at the molecular level. While the experimental evidence from murine and cellular models is compelling, the external validity of these findings requires verification through human clinical studies. Future investigations should explore the effects of AARS1/2 on other proteins and related pathways in addition to the regulation of cGAS, and the stability and specificity of the L-lactate regulatory mechanism in a complex in vivo environment. Additionally, developing targeted inhibitors against AARS1/2 or MCT1 may yield novel therapeutic avenues for immune-related pathologies.

In conclusion, this research establishes AARS1/2 as bifunctional L-lactate sensors and transferases, elucidating their catalytic mechanism in mediating immune regulation (Fig. 1). Interestingly, Li et al. demonstrate that AARS2-mediated cGAS lactylation suppresses innate immune surveillance, providing a mechanistic basis for understanding immune evasion in related pathologies. They also made the seminal discovery that cGAS lactylation mediates anxiety-associated immune dysfunction, revealing a previously unrecognized role for this modification in neuropsychiatric disorders that extends the significance of this mechanism beyond virology and cancer. These findings create a foundation for disease therapeutic development targeting L-lactate metabolism or cGAS lactylation.

## Data Availability

Not applicable.
